# A Case of Abortion, with Treatment

**Published:** 1897-07

**Authors:** J. A. Boyd

**Affiliations:** Thorndale, Texas


For the Texas Medical Journal.

### A CASE OF ABORTION, WITH TREATMENT.

BY J. A. BOYD, M. D., THORNDALE, TEXAS.

ON MAY 19th, 1897, while driving by the house of Mr. B., he asked me to stop to see his wife, who, he said, had been sick for two months. When I went in I found Mrs. B. in bed, where she had been for some time, and succeeded in getting the following history:

Age 18 years, been married 4 years, had one chlid. Up to March, 1897, was in good health. At this time she suffered with a good deal of pain in the region of the uterus, which was partially relieved by what she supposed to be the appearance of her menses. She said that she had been as regular as clock work with her menses all along, and had never suffered any pain before. Her suffering grew more intense within a couple of days, and the flow was so profuse that she concluded to seek medical advice, so Dr. H. was called to see her, and gave her some medicine, as he said, for painful menstruation. He was summoned from time to time, and would as often give medicines of different kinds, until at last, becoming discouraged, she called upon Dr. C., who did not see her, but prescribed medicines to relieve her, though without any good resulting. She said that she had suffered very much for eight weeks, and had grown worse under medical treatment. I was asked to take her case in charge if I thought I could better her condition.

The physical examination revealed a temperature of 99½° F., pulse 100, rather weak, color pale, tongue coated and breath very foul. There was nothing, however, to attract especial attention, except the pain in region of womb, and the hemorrhage.

She only being 18 years old, had not gotten over her false modesty enough to allow me an examination of the womb upon my first visit. I told her very plainly that I had no medicines to give—only to give her twenty-four hours to study over what I thought would be my line of treatment, viz: Local treatment, or, as I thought most probable, curetting.

May 20th, she had a messenger at the gate to ask me to stop again. When I approached her, she said she was willing to be examined, and undergo any treatment I might suggest.

The examination of the womb revealed the following: Body very much enlarged, hard and irregular in outline; cervix enlarged, congested, internal os dilated, with a piece of membrane lodged in the outlet, and blood continually oozing therefrom. My former opinion was then made positive, that she was aborting. It being late in the afternoon, I gave her three doses of bromide of soda, each 20 grains, to be taken during the night, if needed, and asked her to take a large dose of Epson salts by four o'clock on the 21st, and I would be present at eight o'clock to do the operation.

While she objected to the anæsthetic, both because of its danger and because of the extra expense, and knowing the external os to be dilated an inch, or probably more, I agreed to operate without its use, if she would act soldier enough to endure the pain, which she did very nicely.

After cleansing the parts well with a carbolized solution through a speculum, I dilated the os as well as I could, and proceeded to pull away pieces of a macerated fœtus until all large pieces and a perfect, though small, placenta was gotten. A dull curette was then introduced, and the interior of the womb thoroughly scraped. I then allowed the steam of warm carbolized solution to flow through the curette, and wash away all debris as it was detached. From the already congested condition of these parts, I thought best to tampon the uterus, which I did with strips of iodoform gauze. I left her 15 drops of tr. opii, 20 drops of fld. ext. ergot, to be given every two hours, with capsules of 5 gr. each of sulphate of quinine, to be taken three times a day. I did not hear from her until the evening of the 22nd, when I returned and removed the tampon, and gave her another douche, telling her to remain in bed for four or five days. At this time she was almost free from pain, and the flow had ceased altogether. She was getting along nicely, when she was seized by malaria, and had three paroxysms. With this exception, she recovered completely, and has since been in good health, especially so far as her feeling is concerned, but told me that at night she notices her limbs, abdomen and lower eyelids swelling. They swell during the night, but when she rises in the morning and attends to her housework she scarcely can notice any swelling during the day. I have examined one sample of urine, and find no albumen or sugar, specific gravity 1016. I expect to examine another sample of urine soon, but am forced to believe that she having lost blood for two months, left that fluid in such a thin condition as to allow its transudation into the tissues during the quiet hours of rest at night. I have had her upon digitallis and tr. ferri chloride, hoping to be fully convinced that to restore a proper circulation will overcome her trouble, and we will not find what seems so suspicious, viz: a kidney lesion.

Should we find albumen, we will then have discovered the cause of all her trouble, or, at least, will have a probable cause, but now we cannot assign the result to any sure cause, as she was in good health, and never knew she was pregnant.

